# Supplementary figures and images for: ADAGE signature analysis: differential expression analysis with data-defined gene sets
Source: BMC Bioinformatics. 2017 Nov 22;18:512. doi: 10.1186/s12859-017-1905-4 (PMC5700673; doi:10.1186/s12859-017-1905-4)

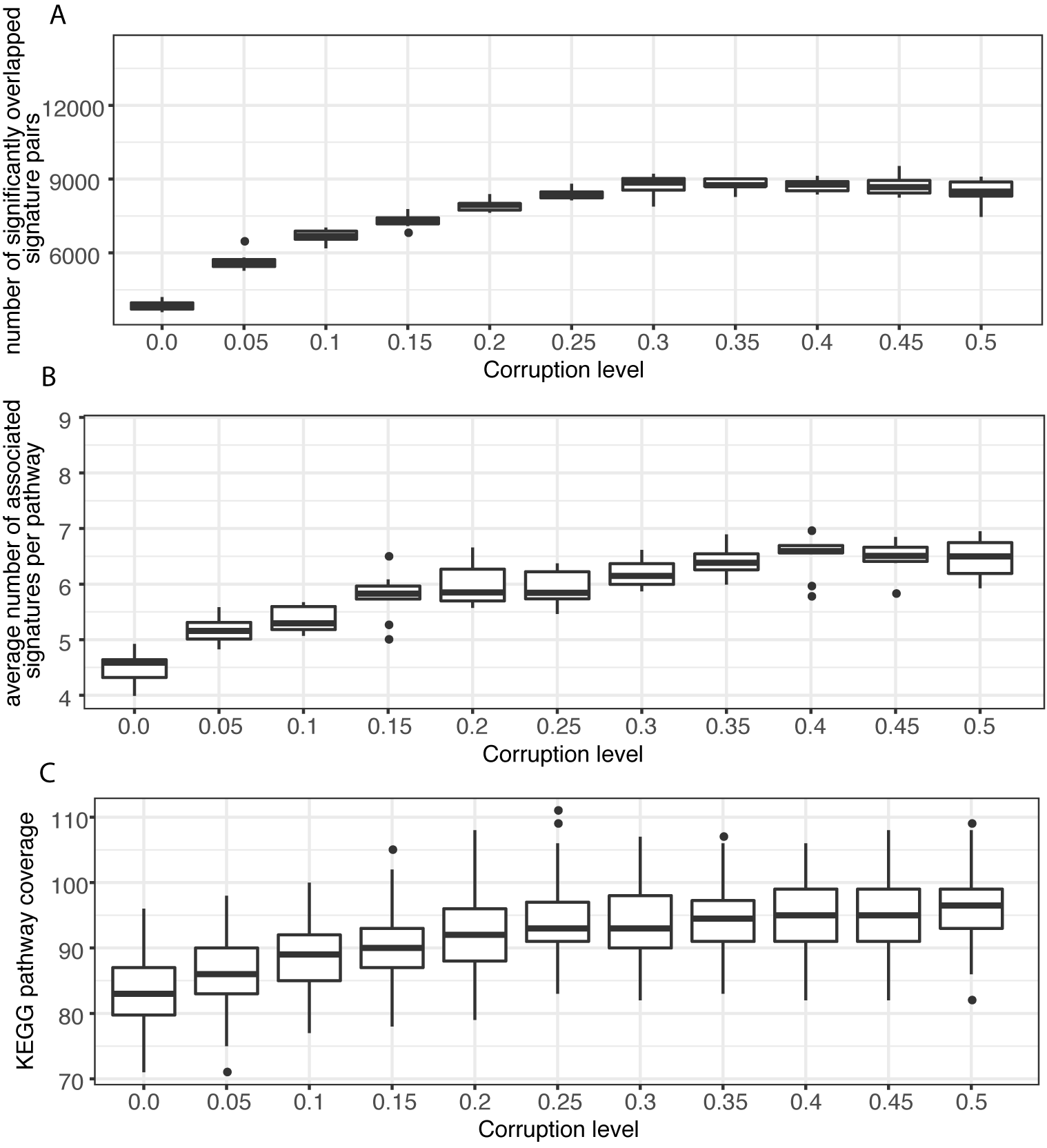

Supplement: Supplementary file 1 — The relationship between corruption level used in building ADAGE models and the redundancy of signatures derived from the models. The plot summarized results from 100 ADAGE models built at each corruption level. A: The number of signature pairs with gene compositions significantly overlapped increases with corruption level until the corruption level reaches 30%. B: As corruption level increases, the number of signatures in a model that enriched of the same KEGG pathway also increases on average, indicating the signatures become more redundant. C: ADAGE models tend to capture more unique KEGG pathways (pathway coverage) when more noise was added during training until the corruption level is higher than 25%. (TIFF 903 kb) [file 12859_2017_1905_MOESM1_ESM.tif]

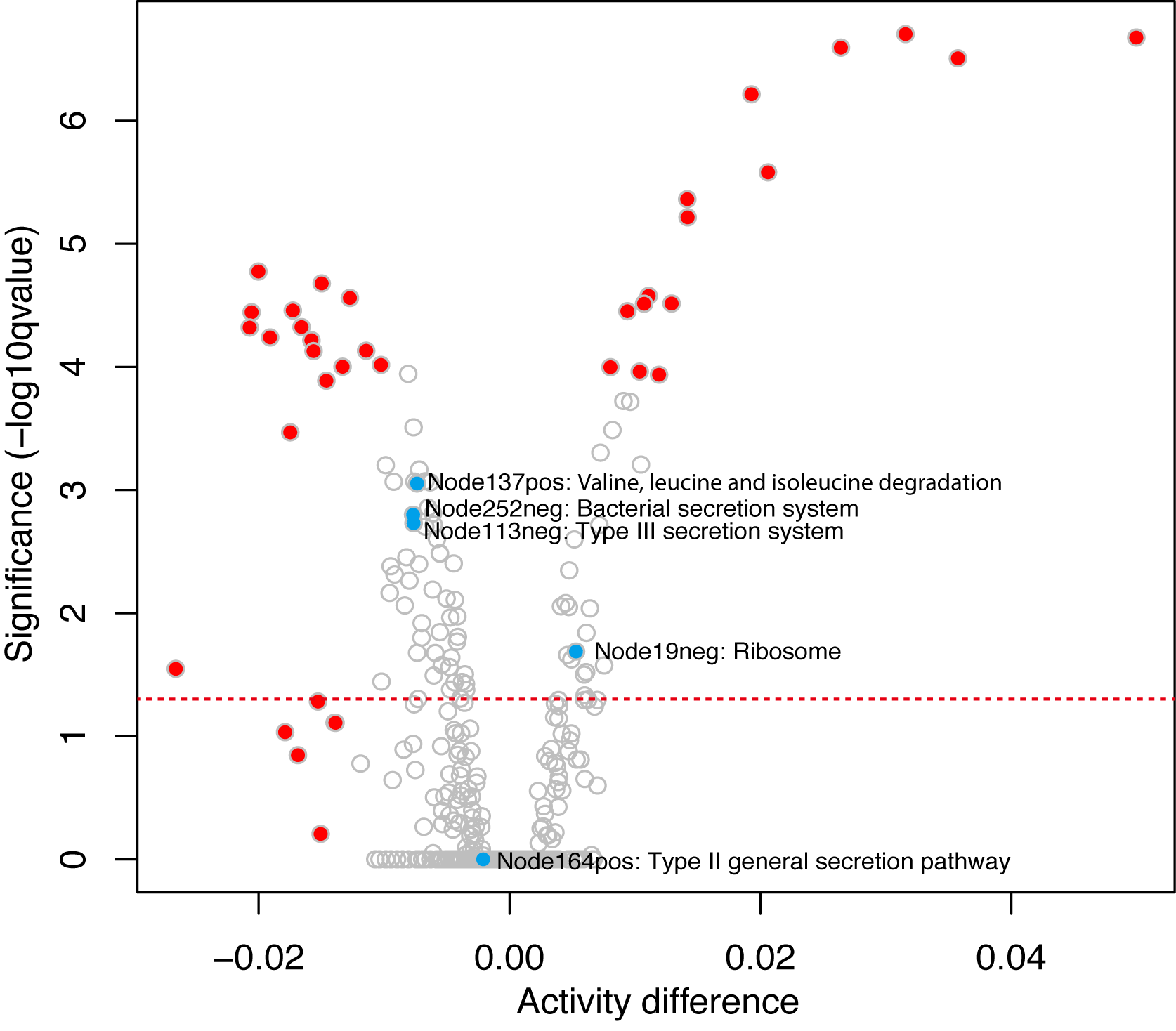

Supplement: Supplementary file 2 — KEGG pathways enriched in the GSEA analysis but not associated with selected signatures in the ADAGE signature analysis. In the same volcano plot as Fig. 4a, signatures associated with GSEA-only pathways are highlighted in blue while signatures lie on the first 10 Pareto fronts are highlighted in Red. Node137pos, Node252neg, and Node113neg obtained high significances in the activation test and would be considered if we lose the activation cutoff. Pathways Tryptophan biosynthesis, chorismate = > tryptophan (KEGG-Module-M00023) and Aminoacyl-trna biosynthesis (KEGG-Pathway-pae00970) are not associated with any signature, so they were not labeled in the plot. (TIFF 746 kb) [file 12859_2017_1905_MOESM2_ESM.tif]

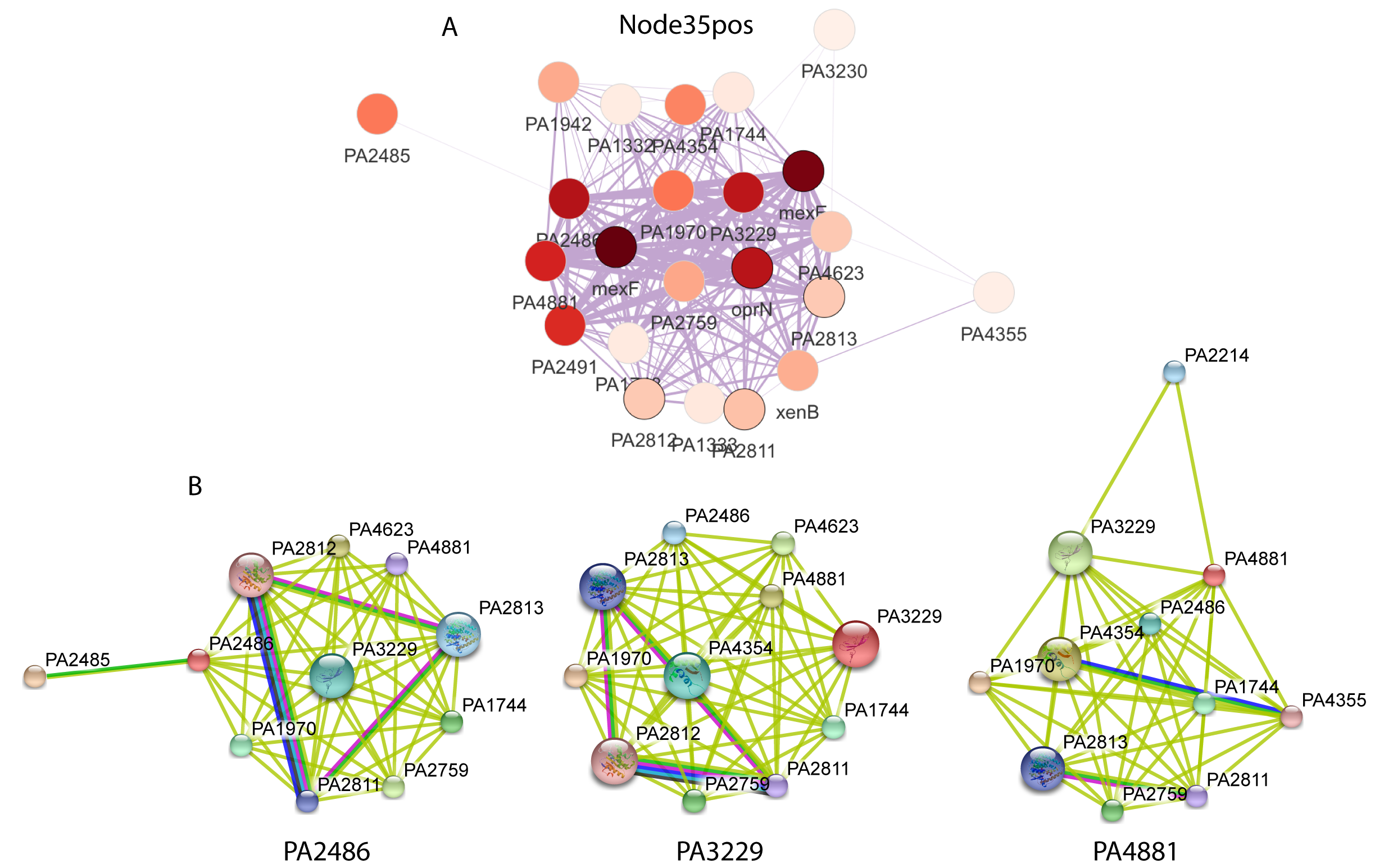

Supplement: Supplementary file 3 — Validation of Node35pos as a transcriptional program via the STRING network. A: The largest connected module of the gene-gene network subset by genes in Node35pos. B: Gene-gene networks returned by STRING when searching PA2486, PA3229, and PA4881 respectively. The STRING networks of the three genes are subsets of the Node35pos network. (TIFF 2780 kb) [file 12859_2017_1905_MOESM3_ESM.tif]

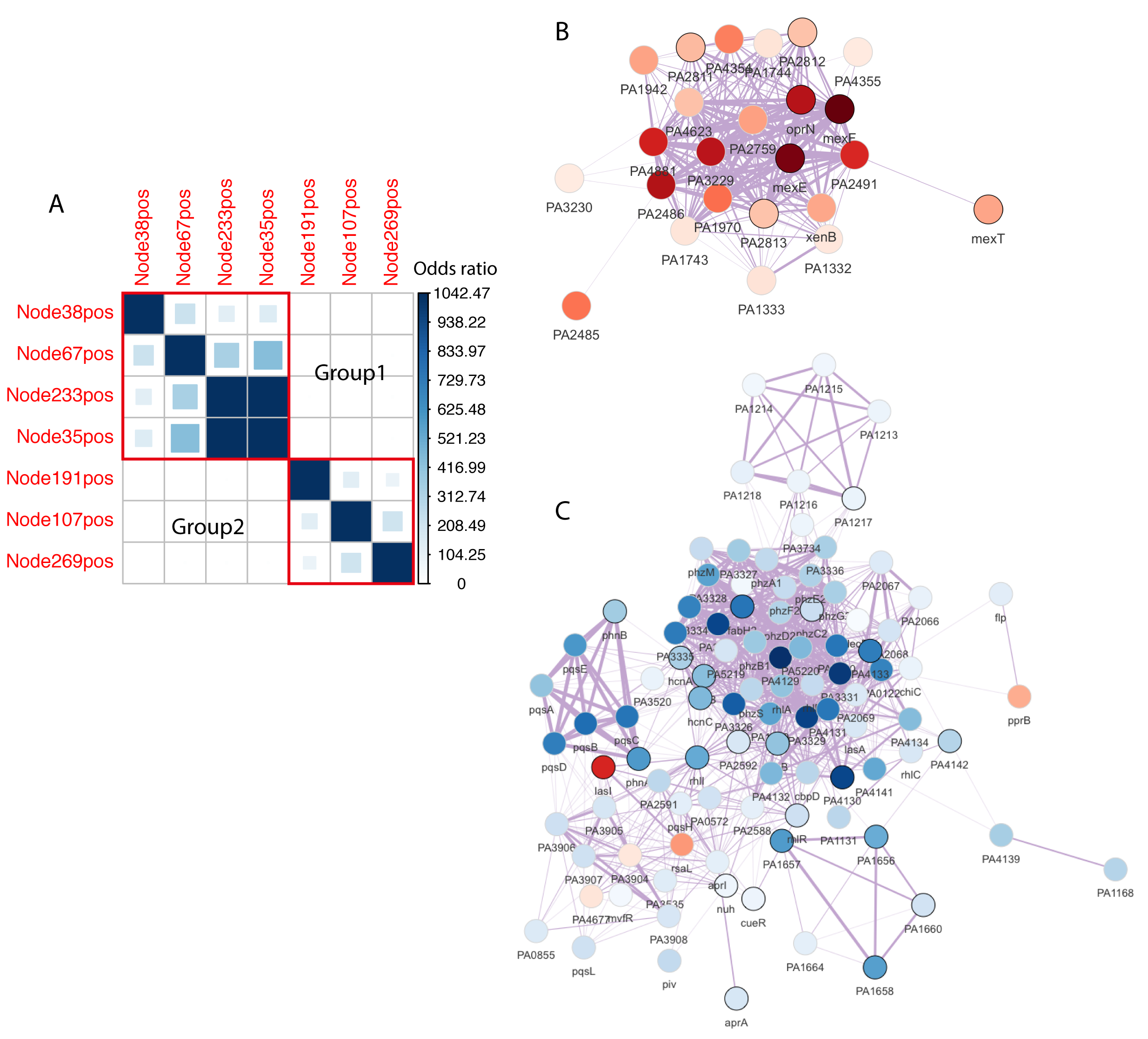

Supplement: Supplementary file 4 — Groups of signatures that are uncharacterized by KEGG. A: The signature similarity heatmap of uncharacterized signatures. Heatmap color reflects the odds ratio that two signatures overlap in their gene contents. Signatures are divided into two groups based on their similarity. B: The largest connected module in the gene-gene network subset by genes in Group1 signatures. This module contains the MexT regulatory program. C: The largest connected module in the gene-gene network subset by genes in Group2 signatures. This module contains many genes involved in quorum sensing. (TIFF 3586 kb) [file 12859_2017_1905_MOESM4_ESM.tif]
